# Broadly Applicable, Virus-Free Dual Reporter Assay to Identify Compounds Interfering with Membrane Fusion: Performance for HSV-1 and SARS-CoV-2

**DOI:** 10.3390/v14071354

**Published:** 2022-06-21

**Authors:** Nica Classen, Diana Ulrich, Arne Hofemeier, Marc Tim Hennies, Wali Hafezi, Aleksandra Pettke, Marie-Luise Romberg, Eva U. Lorentzen, Andreas Hensel, Joachim E. Kühn

**Affiliations:** 1 Institute of Pharmaceutical Biology and Phytochemistry, University of Münster, Corrensstr. 48, 48149 Münster, Germany; n.classen@uni-muenster.de (N.C.); diana.ulrich@hotmail.de (D.U.); ahensel@uni-muenster.de (A.H.); 2 Institute of Virology—Clinical Virology, University of Münster and University Hospital Münster, Von-Stauffenberg-Str. 36, 48151 Münster, Germany; arne.hofemeier@phys.uni-goettingen.de (A.H.); marctim.hennies@ukmuenster.de (M.T.H.); hafezi@uni-muenster.de (W.H.); aleksandra.pettke@folkhalsomyndigheten.se (A.P.); marie-luise.romberg@ukmuenster.de (M.-L.R.); eva.lorentzen@ukmuenster.de (E.U.L.); 3 Vibalogics GmbH, Zeppelinstr. 2, 27472 Cuxhaven, Germany; 4 Third Institute of Physics—Biophysics, University of Göttingen, Friedrich-Hund-Platz 1, 37077 Göttingen, Germany; 5 The Public Health Agency of Sweden, Nobels väg 18, 171 82 Solna, Sweden

**Keywords:** cell–cell fusion, HSV-1, SARS-CoV-2, fusion inhibition, dual reporter assay, secreted alkaline phosphatase, secreted nanoluciferase

## Abstract

Membrane fusion constitutes an essential step in the replication cycle of numerous viral pathogens, hence it represents an important druggable target. In the present study, we established a virus-free, stable reporter fusion inhibition assay (SRFIA) specifically designed to identify compounds interfering with virus-induced membrane fusion. The dual reporter assay is based on two stable Vero cell lines harboring the third-generation tetracycline (Tet3G) transactivator and a bicistronic reporter gene cassette under the control of the tetracycline responsive element (TRE3G), respectively. Cell–cell fusion by the transient transfection of viral fusogens in the presence of doxycycline results in the expression of the reporter enzyme secreted alkaline phosphatase (SEAP) and the fluorescent nuclear localization marker EYFPNuc. A constitutively expressed, secreted form of nanoluciferase (secNLuc) functioned as the internal control. The performance of the SRFIA was tested for the quantification of SARS-CoV-2- and HSV-1-induced cell–cell fusion, respectively, showing high sensitivity and specificity, as well as the reliable identification of known fusion inhibitors. Parallel quantification of secNLuc enabled the detection of cytotoxic compounds or insufficient transfection efficacy. In conclusion, the SRFIA reported here is well suited for high-throughput screening for new antiviral agents and essentially will be applicable to all viral fusogens causing cell–cell fusion in Vero cells.

## 1. Introduction

In enveloped viruses, entry into host cells is executed by specialized surface proteins mediating fusion between viral and cellular membranes (reviewed by [1,2,3,4]). Hence, the membrane fusion machinery is indispensable in the replication cycle of enveloped viruses and represents an important druggable target. Fusion-inhibiting compounds have been developed and approved in the therapy of a number of viral infections (reviewed by [5,6]). For example, enfuvirtide, a fusion inhibitor targeting the heptad repeat region of human immunodeficiency virus type 1 (HIV-1) glycoprotein gp41, is used successfully in combination with other antiretroviral drugs as a second-line therapeutic against drug-resistant strains [5]. In addition to viral fusion proteins, virus entry-related targets may comprise cellular targets, e.g., biomembranes or cellular proteases priming membrane fusion [7,8].

The activity of viral fusion proteins can induce the formation of large polykarya termed syncytia (reviewed by [9]). Syncytia formation represents a special form of virus-induced membrane fusion, which occurs in various viruses, tissues and cell types in vitro and in vivo, and may be involved in the molecular pathogenesis of viral infections [10] for severe acute respiratory syndrome coronavirus 2 (SARS-CoV-2) reviewed by [11].

Usually, the molecular mechanisms of membrane fusion underlying syncytia formation and virus entry largely overlap. Depending on the viral agent, the expression of viral fusion proteins (fusogens) on the cell surface in the absence of an infectious virus may also result in cell–cell fusion and syncytia formation (reviewed by [4]). In this case, quantifying cell–cell fusion enables the determination of viral fusion activity in the absence of infectious particles and hence circumvents biosafety issues encountered when working with infectious, pathogenic viruses.

A first pioneering approach to quantifying cell–cell fusion in a virus-free assay was published by [12] for the human T-lymphotropic virus 1 (HTLV-1) fusion machinery. The assay is based on the measurement of luciferase expression under the control of a T7 promoter, which is only activated in case of membrane fusion with a neighboring cell expressing the T7 polymerase. In the following, T7-polymerase/promoter-based assays were established and optimized for various viruses. Besides luciferase, reporter genes such as green fluorescent protein (GFP) or beta-galactosidase were applied [13,14]. Luciferase-based assays have been used to study fusion by herpes simplex virus type 1 (HSV-1), e.g., by applying a T7 readout system [12]. Furthermore, the expression of reporter genes as split-domain proteins showing activity only when both domains are present in a fused cell [15,16] enables measurement of fusion kinetics. For example, a cell-based assay for the quantification of SARS-CoV-2 spike-mediated fusion activity using the HIV-1 Tat promoter in combination with the LTR-luciferase gene has been published by [17] for drug discovery purposes. Another approach established by Thakur et al. (2021) [18] depending on split-domain GFP-*Renilla* luciferase as the reporter enzyme, aims at detecting neutralizing antibodies.

The limitations of cell–cell fusion assay systems developed so far include highly sophisticated workflows and a multiplicity of subsequent steps, potentially leading to high inter-assay variability. In addition, the insufficient stability of reporter systems and inadvertent impacts of assay procedures on cell behavior such as the stimulation of the innate immune responses have compromised past approaches. Despite a variety of improvements, there still appears to be room for the methodical refinement of reporter assays quantifying viral fusion activity. The aim of the present study was to establish a robust, sensitive and specific virus-free reporter fusion assay with the main focus on compound screening. Thus, the emphasis was laid on its broad applicability for different viruses, simple handling, modular set-up, possibility of repeated measurements and reliable discrimination of true hits from false hits due to cytotoxic effects. Reporter gene expression controlled by the powerful third-generation tetracycline-inducible Tet3G-On system, consisting of the tet responsive element (TRE3G) and the transactivator (Tet3G), was used to exclude any pronounced effect of hypericin on SARS-CoV-2-induced cell–cell fusion [19]. Here, we report the systematic improvement of this assay, which led to the establishment of a dual reporter gene assay for the screening and identification of antifusogenic compounds. The assay was based on the fusion-specific induction of the secreted reporter alkaline phosphatase (SEAP) in complementary stable Vero cell lines by the Tet3G-On system, and secreted nanoluciferase (secNLuc) as the internal reporter control.

Although membrane fusion shares common properties and mechanisms that appear to be ultimately conserved, viral fusion machineries offer a stunning diversity with respect to complexity, the need for accessory proteins, and viral and cellular factors triggering the fusion event (reviewed by [4]). Structural and functional similarities have led to the classification of viral fusion proteins into three classes (reviewed by [20]). The performance of the reporter fusion assay reported here was tested with two different, prototypic, pH-independent class I and class III fusion machineries, i.e., SARS-CoV-2 and HSV-1, respectively.

For SARS-CoV-2, a betacoronavirus of the family *coronaviridae* and causative agent of the coronavirus disease 2019 (COVID-19) pandemic, the spike protein (S), located in the viral membrane as a homotrimer, functions as the fusion protein, as also observed for other coronaviruses [21,22]. The receptor binding domain (RBD) of the spike protein binds to the human host cell receptor protein angiotensin converting enzyme 2 (ACE2) [23] and fusion is triggered either by TMPRSS2 priming, or by cathepsins B and L via the endosomal pathway [7]. During SARS-CoV-2 infection, syncytia are formed by epithelial cells [24], and the spike-mediated vertical virus spread by cell–cell transmission may play a role in the pathomechanism of COVID-19 [25].

HSV-1, a member of the family *herpesviridae*, contains a complex fusion machinery. The following four glycoproteins are essential for membrane fusion: gB, gD, and the heterodimer gH/gL [26,27]. The fusion mechanism of HSV-1 depends on the binding of gD to a cell receptor such as nectin-1 or HVEM [28,29], and transfer of the signal to the gH/gL heterocomplex, which finally activates the viral fusogen gB [30,31].

Our data show that the stable reporter fusion inhibition assay (SRFIA) presented here is well suited for the quantification of virus-induced membrane fusion. We proved the functionality of this assay for the HSV-1 and SARS-CoV-2 fusion machineries, respectively, and developed a method to determine cell viability and transfection efficiency by the co-transfection and quantification of secNLuc in parallel. Its modular set-up provides the high-throughput screening (HTS) of compounds with putative antifusogenic properties. In principle, the SRFIA reported here will be applicable to all viral fusogens causing cell–cell fusion in Vero cells.

## 2. Materials and Methods

### 2.1. Plasmids

Plasmids expressing HSV-1 glycoproteins: Expression plasmids pPEP99, pPEP100 and pPEP101 containing HSV-1 glycoproteins D, H and L, respectively, were a kind gift from Anthony Nicola, Washington State University, Pullman, WA, USA [27].

Plasmid expressing SARS-CoV-2 spike protein: Expression plasmid pCG1-SARS-2-S encoding the Wuhan strain SARS-CoV-2 S protein was kindly provided by Stefan Pöhlmann, German Primate Center, Göttingen, Germany [7].

Cloning of wt gB-1: Using primers gBXX-Bgl fw (5′-GCAGATCTCGTAGTCCCGCCATGCGCCAGG-3′), gBXX-Spe-Kpn bw (5′-GCGGTACCGAATTCACTAGTAGACCCACGGCCAGC GCCC-3′) and Q5 DNA polymerase (New England Biolabs GmbH, Frankfurt/Main, Germany), a 2385 bp long PCR fragment containing the ectodomain and parts of the transmembrane domain of gB was amplified from the vector pKBXX (kindly provided by Roberto Manservigi, University of Ferrara, Ferrara, Italy), [32,33]. Primer gBXX-Spe-Kpn bw introduced three silent mutations in gB-1 at positions 2346 (G > A), 2347 (T > C) and 2349 (G > A) to create a unique SpeI site. The missing C-terminal parts of gB-1 were generated as a 380 bp PCR product with the primers gBXX-Spe fw (5′-GCA CTA GTC CTG GCC GGC CTG GC-3′) and gBXX-Kpn bw (5′-GCG GTA CCT CAC AGG TCG TCC TCG TCG GCG TC-3′) using Q5 DNA polymerase and purified HSV-1 17 DNA as the target sequence. Both PCR fragments were cloned consecutively into the eukaryotic expression vector pBacMam-2 (Novagen, Merck-Millipore, Darmstadt, Germany) via BglII/KpnI and SpeI/KpnI, respectively. The final construct was termed pBacgBXX-Spe. Primers were delivered by Biomers, Ulm, Germany, and restriction enzymes by New England Biolabs.

Cloning of pEN-secNL: Vector pEN-secNL encoding a constitutively expressed, secreted form of nanoluciferase (NLuc) was generated by amplifying NLuc sequences of vector pNL1.3 (Promega, Walldorf, Germany) by PCR with primers secNL fwd (5′-CGT CAG ATC CGC TAG CAT GAA CTC CTT CTC CAC AAG C-3′) and NL bwd (5′-TCT AGA GTC GCG GCC GCT TAC GCC AGA ATG CGT TCG C-3′) and inserting the product by In-Fusion^TM^ (Clontech, Mountain View, CA, USA) cloning into pEGFP-N1 (Clontech) opened with NheI and NotI. All cloned plasmid inserts were confirmed by sequencing (Eurofins, Ebersberg, Germany).

### 2.2. Cell Culture

Vero E6 and BHK-21 cells were cultivated as described earlier [34]. MEM Eagle (1×) supplemented with 10% FCS, 1% L-glutamine 200 mM, 1% penicillin/streptomycin solution (10,000 U/10,000 µg/mL) and 1% NEA solution (100×), all purchased from Sigma-Aldrich/Merck, Darmstadt, Germany, were used as the standard cell culture medium if not stated otherwise.

### 2.3. Stable Reporter Cell Lines

Vero CMVTet3G. The stable cell line Vero CMVTet3G was generated by transfecting Vero E6 cells with the vector pCMV-Tet3G (Clontech) and selecting G418-resistant clones stably expressing the Tet3G transactivator.

Vero TRE3G-SEAP-EYFPNuc. The stable cell line Vero TRE3G-SEAP-EYFPNuc expressing SEAP and EYFP with a nuclear localization signal (EYFPNuc) from a bicistronic transcript under the control of the TRE3G promoter was generated as follows. The EYFPNuc coding sequences of plasmid pEYFPNuc (Clontech) were amplified with primers EYFPNuc fw and EYFPNuc bw (5′-GCC GGA TAT CAC GCG TAT GGT GAG CAA GGG CGA G-3′, 5′-ATC CCT GCA GGC TAG CTT ATC TAG ATC CGG TGG ATC CTA C-3′) and inserted by In-Fusion^TM^ cloning into the vector pTRE3G-ZsGreen1 (Clontech) opened with MluI and NheI. Next, the ZsGreen1 coding sequence was excised with SalI and FseI and replaced by SEAP amplified from the vector pSEAP2-basic (Clontech) using primers SEAP-fw and SEAP-bw (5′-CCC TCG TAA AGT CGA CAT GCT GCT GCT GCT GCT GCT GCT G-3′, 5′-AGG GAG AGG GGC CGG CCT CAT GTC TGC TCG AAG CGG CCG GC-3′) and In-Fusion cloning for the insertion of the PCR fragment. After linearization with ScaI, pTRE3G-SEAP-EYFPNuc was cotransfected with linear hygromycin marker (Clontech) into Vero E6 cells. Cells were grown in 24-well plates overnight in the absence of a selection marker, reseeded into 6-well plates, cultivated in the presence of 500 µg/mL hygromycin B (Invitrogen, Carlsbad, CA, USA) until confluency was reached, and split at a 1:1 ratio. One half of the cells was transfected with pCMV-Tet3G, the other served as the negative control. Both populations were trypsinized (trypsin/EDTA solution 0.05%/0.02% (Merck) in PBS) 24 h later, resuspended in PBS (Biochrom, Berlin, Germany) supplemented with 10% FCS (Biochrom), and pelleted at 1000× *g*. Approximately 10^6^ cells were resuspended in 1000 µL PBS containing 30 µL trypsin/EDTA solution. The cells were analysed using FACS Aria III (BD Biosciences, Franklin Lakes, USA) gated for the untransfected cell population with the software FACS Diva Version 6.1.3 and sorted for fluorescence intensity of EYFPNuc. Single cells with high fluorescence were sorted into a 96-well plate containing a culture medium with 500 µg/mL hygromycin B and 1% (*v*/*v*) antibiotic/antimycotic (Fisher Scientific, Schwerte, Germany). Clones were allowed to grow for approximately two weeks, wells containing a single clone were further expanded and tested for reporter gene expression by transfection with pCMV-Tet3G and the addition of 10 µg/mL doxycycline.

### 2.4. Stable Reporter Fusion Assay (SRFIA) with HSV-1 Fusion Machinery

Vero CMVTet3G (Vero CMV) expressing the Tet3G transactivator and Vero TRE3G-SEAP-EYFPNuc (Vero SEAP) containing a bicistronic SEAP-EYFPNuc reporter gene under the control of the TRE3G responsive element were cultivated in standard medium in the presence of the appropriate selection markers, e.g., G418 and hygromycin B, respectively. Prior to the preparation of the reporter cell mixture, cells were cultivated for 24 h in the absence of selection markers. Subsequently, reporter cells were resuspended and mixed in equal parts. A total of 1.25 × 10^5^ cells/well were seeded into 24-well culture plates and cultivated for 20 h in standard medium. Cells were transfected with the HSV-1 fusion machinery using 500 ng of pBacgBXX-Spe, and 660 ng of pPEP99, pPEP100, pPEP101, respectively, 1 µL Lipofectamine™ 2000 reagent and 260 µL Opti-MEM medium unless otherwise indicated. For mock transfection, 500 ng of the empty vector backbone pBacMam-2 was transfected instead of pBacgBXX-Spe. Then, 3.5 h after transfection, supernatants were discarded, cells were washed once with PBS and overlaid with 1 mL MEM 10% FCS supplemented with doxycycline (10 µg/mL) and substances were tested at different concentrations. 1-Docosanol was solved as described by [35] with Pluronic-F-68 (Sigma-Aldrich), *aqua ad iniectabilia* (B. Braun, Melsungen, Germany) and MEM-Earle’s (2×), supplemented with L-glutamine, at a ratio of 1:9:10. Monoclonal antibody MAb 2c was kindly provided by Anna Maria Eis-Hübinger [36]. SEAP activity was determined 48 h post-transfection at 37 °C, 5% CO_2_ with the Phospha-Light SEAP Reporter Gene System™ (Fisher Scientific) according to the manufacturer´s instructions. A total of 100 µL of cell culture supernatant was mixed with 100 µL dilution buffer (1×) in 1.5 mL reaction tubes and incubated at 65 °C for 30 min to suppress endogenous phosphatase activity. After cooling on ice to room temperature, 100 µL of the diluted supernatant was mixed with 100 µL assay buffer and incubated for 5 min at room temperature. Afterwards, the 100 µL reaction buffer was added and samples were incubated for 15 min at room temperature in the dark. Chemiluminescence was quantified in a GloMax^®^ 20/20 luminometer (Promega) with an integration time of 1 s. Cell viability was determined using the XTT assay from the same well as described below. Each measuring point was run at least in technical triplicates.

To transfer the assay to the 96-well format, 6 × 10^5^ cells/well of each cell line were seeded into 6-well plates and incubated for 20 h. Transfection was carried out using 1.5 µg pBacgBXX-Spe, 1.98 µg pPEP99, pPEP100 and pPEP101, respectively, and 700 µL Opti-MEM per well. A total of 4 µL of the Lipofectamine™ 2000 reagent was added. After 4 h of incubation, cells were washed with PBS once and trypsinized with 300 µL of trypsin/EDTA solution. Cells were counted and a quantity of 7.5 × 10^4^ cells/well was seeded into 96-well culture plates. A standard medium containing test substances was added and cells were incubated for 48 h, 37 °C, 5% CO_2_. The SEAP measurement was performed as described above except for using only 50 µL of supernatant and reagents.

### 2.5. Stable Reporter Fusion Assay (SRFIA) with SARS-CoV-2 Fusion Machinery

For the SARS-CoV-2 S protein-mediated fusion assay, cell lines were prepared analogous to the HSV-1 fusion assay. The 2 × 10^5^ cells were seeded into 24-well culture plates and incubated for 20 h. Cells were transfected with 2 µL of Lipofectamine^TM^ 2000 reagent, 250 ng of pCG1-SARS-2-S vector plasmid and 320 µL Opti-MEM medium. After 4 h of incubation and PBS washing, cells were overlaid with 500 µL of MEM/10% FCS supplemented with doxycycline (10 µg/mL) and umifenovir hydrochloride (Sigma-Aldrich/Merck) as positive controls in different concentrations solved in DMSO or DMSO only as the untreated control. SEAP expression levels were quantified after 24 h of incubation with the Phospha-Light SEAP Reporter Gene System^TM^ as described for HSV-1 except with the use of 50 µL of supernatant and reagents. The determination of SEAP levels was carried out on white 96-well plates (Greiner Bio-One, Frickenhausen, Germany) and measurements were performed using a GloMax^®^ Explorer luminometer (Promega) with an integration time of 1 s.

To perform the assay on 96-well plates, 6 × 10^5^ cells of each cell line were seeded into 6-well plates. After 20 h of incubation, 750 ng of pCG1-SARS-2-S was transfected with 4 µL Lipofectamine™ 2000 reagent. Following 3.5 h of incubation, cells were washed with PBS once, resuspended by adding 300 µL of trypsin/EDTA solution and collected. Subsequently, cell numbers were determined. Then, 7.5 × 10^4^ cells were seeded into each well of the 96-well plate after adding doxycycline (10 µg/mL) to the cell suspension. MEM FCS 10% was added to a final volume of 150 µL, containing test substances or DMSO as the vehicle control. Cells were incubated for 48 h.

For time kinetic measurement, volume reduction was considered by using the following formula:

$$RLU\left(corr\right)=RLU_{t1}*\left(\frac{V\left(sample1\right)}{V\left(original\right)}\right)+\ldots +RLU*\left(\frac{V\left(remaining\right)}{V\left(original\right)}\right)$$

### 2.6. Measurement of NLuc Activity

On 24-well plates, a 1 ng pEN-secNL vector was co-transfected with plasmids for the fusion machinery. For HTS on 96-well plates, 3 ng of plasmid was used for the transfection of 6-well plates. Activity of secNLuc was determined with the NanoGlo^®^ Luciferase assay system (Promega) [36]. For assays on 24-well plates as well as on 96-well plates after 24 or 48 h of incubation, respectively, the secreted NL activity was determined using cell culture supernatant according to the manufacturer’s instruction. A total of 20 µL of supernatant was diluted with 80 µL deionized water on a black 96-well culture plate. The assay reagent was prepared according to the manufacturer’s instructions by mixing the substrate solution and assay buffer at a ratio of 1:50. After adding 20 µL of the assay reagent, chemiluminescence was quantified in a GloMax^®^ Explorer with an integration time of 0.3 s.

### 2.7. Cytotoxicity Assay XTT

The XTT assay was performed after 24 or 48 h of incubation, respectively, directly on cell cultures with the remaining supernatant after taking samples for the SEAP and secNLuc assay. For the determination of cell viability, the ab 232,856 XTT assay kit (abcam, Cambridge, UK) was used. According to the manufacturer’s instruction, 10 µL of the XTT mixture was added to culture wells and incubated for 2 h at 37 °C, 5% CO_2_. Absorbance was determined at λ = 450 nm in a GloMax^®^ Explorer plate luminometer. Untreated transfected controls served as the 100% reference.

### 2.8. Microscopical Determination of Syncytia Formation

Microscopical pictures of cell–cell fusion were taken with an Axiovert 200 fluorescence microscope with a Colibri 7 light source, Axiocam 512 mono, and data analysis and controlling were performed using Zen software (Zeiss, Jena, Germany).

Microscopical pictures of SARS-CoV-2 S mediated cell–cell fusion were obtained by fixing cells with 3.7% formaldehyde, permeabilization by Triton X-100 0.5% in PBS and staining with human anti-S polyclonal serum (1:100), goat-anti-human F(ab)_2_ biotin (1:500; Dianova), streptavidin-FITC (1:500; Life technologies,) and 4′,6-diamidin-2-phenylindol (1:1000; Sigma-Aldrich).

Following methanol fixation, the Giemsa-staining of cells was performed using Giemsa staining solution (Sigma-Aldrich/Merck) according to the manufacturer’s instructions.

### 2.9. Calculation of Zhang Indices

Values were calculated following [37] to assess the suitability of the assay method as HTS. Following Zhang et al., the *Z* value provides information about separation of data variation bands, indicating the ability of the assay to detect real hits.

The used formula is as follows:

$$Z=1-\frac{\left(\;3SD_{sample}+3SD_{control}\right)}{\left(\left|mean\;sample-mean\;control\right|\right)}$$

For calculation, the log10 transformed values were used.

### 2.10. Statistics

An unpaired two-tailed student’s t-test was used for the comparison against the controls for single group comparison, and for multiple group comparison, a one-way ANOVA test followed by the Bonferroni-test in case of significant differences with confidence intervals of 95% was performed using GraphPad Prism Software Version 3.0 (GraphPad Software, San Diego, CA, USA) with *p* < 0.05 considered as statistically significant (*), *p* < 0.01 considered as statistically very significant (**) and *p* < 0.001 considered as statistically highly significant (***). Experiments were performed in at least technical triplicates. EC50 values were also calculated using a nonlinear regression analysis (curve fit) in GraphPad Prism.

## 3. Results

### 3.1. Bicistronic Reporter Gene Cassette and Stable Vero Reporter Cell Lines

**Figure 1:**
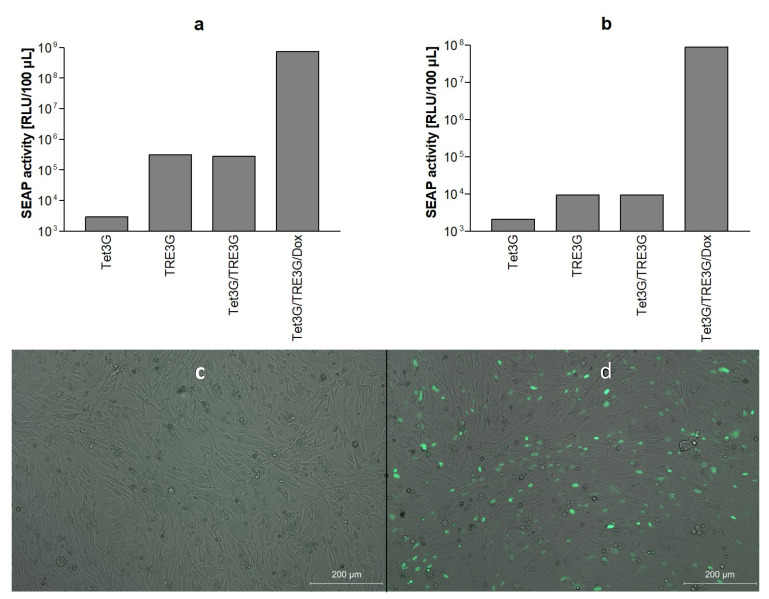
Function of bicistronic SEAP/EYFPNuc reporter gene cassette. Cells cultivated in 24-well plates were transiently transfected with transactivator Tet3G plasmid pCMV-Tet3G (Tet3G), reporter gene plasmid pTRE3G-SEAP-EYFPNuc (TRE3G), both (Tet3G/TRE3G) or both treated with doxycycline (Tet3G/TRE3G/Dox) as indicated, using 100 ng plasmid, respectively, and SEAP secreted into culture supernatants was quantified 24 h later, panel (**a**): BHK-21 cells, panel (**b**): Vero E6 cells. Induction of EYFPNuc expression in BHK-21 cells co-transfected with pCMV-Tet3G and pTRE3G-SEAP-EYFPNuc in the absence (**c**) or presence (**d**) of 10 µM doxycycline was analyzed by fluorescence microscopy (transmission light microscopy of intact cells overlayed by EGFP-fluorescence).

The bicistronic reporter plasmid pTRE3G-SEAP-EYFPNuc which provides the simultaneous expression of SEAP and EYFPNuc was cloned as described in the Materials and Methods. In the presence of doxycycline, the expression of the SEAP reporter gene was found to be strongly induced by co-transfection with vector pCMV-Tet3G encoding the tet transactivator (Figure 1a,b).

The inducible expression of the fluorescent subcellular localization marker EYFPNuc by the bicistronic reporter construct was controlled microscopically. The transient co-transfection of cells with pCMV-Tet3G and pTRE3G-SEAP-EYFPNuc caused the prominent fluorescent staining of nuclei in the presence of doxycycline 24 h after transfection in BHK-21 (Figure 1c,d) and Vero E6 cells (data not shown).

Having shown that the bicistronic tet-responsive reporter system provides the inducible expression of SEAP and EYFPNuc in transient transfection assays, stable Vero cell lines containing the individual components of the bipartite tet-responsive reporter system were generated as given in the Materials and Methods.

**Figure 2:**
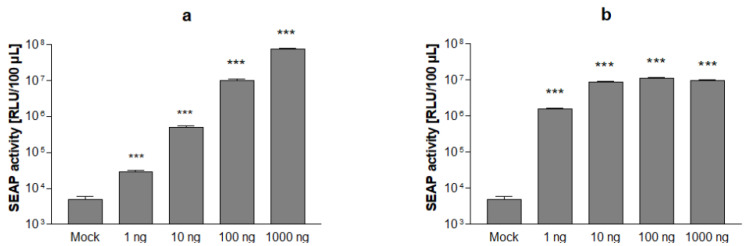
Induction of SEAP expression in Vero indicator cells. Stable cell lines Vero CMVTet3G and Vero TRE3G-SEAP-EYFPNuc mixed at a 1:1 ratio and cultivated in 24-well plates were transiently transfected with pTRE3G-SEAP-EYFPNuc (**a**) and pCMV-Tet3G (**b**) as indicated. SEAP levels in culture supernatants after cultivation in the presence of 10 µg/mL doxycycline for 48 h are shown. Data represent means ± SD from quadruplicates. Significance is indicated compared to non-transfected control (Mock), *p* < 0.001 (***).

After clonal expansion and serial passaging, two stable cell lines termed Vero CMVTet3G and Vero TRE3-SEAP-EYFPNuc, respectively, were chosen for further experiments. In the presence of doxycycline, the transfection of less than 1 ng plasmid DNA carrying the tet transactivator or the tet-responsive reporter gene cassette, respectively, was found to be sufficient to induce significant SEAP expression above the background level in stable Vero reporter cells mixed in equal parts and seeded into 24-well plates (Figure 2a,b).

### 3.2. HSV-1 Fusion Machinery Induces Syncytia in Vero Reporter Cells

**Figure 3:**
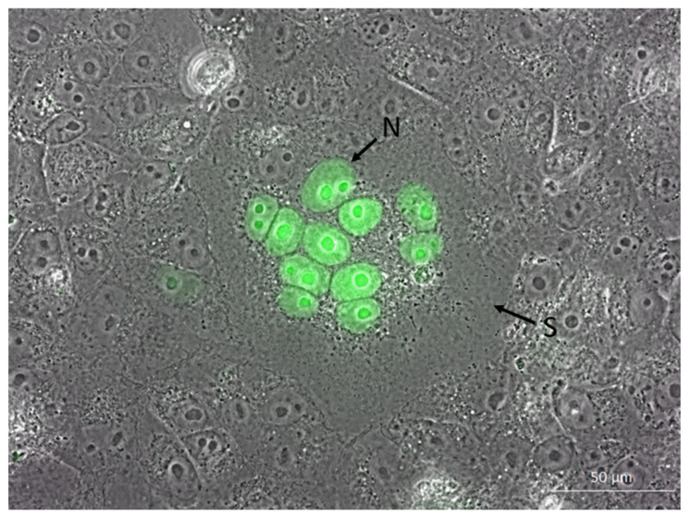
Induction of syncytia formation by the HSV-1 fusion machinery in Vero reporter cells. Transmission light microscopy of intact cells with phase contrast overlayed with EGFP-fluorescence, 63× oil. Green: EYFPNuc-positive nuclei. Arrows indicate syncytium (S) and nuclei (N).

We next studied whether the transfection of Vero E6 reporter cells with the HSV-1 core fusion machinery, i.e., glycoproteins B, D, H and L, results in the formation of syncytia as observed in parental Vero E6 cells [27]. As seen in Figure 3, stable Vero reporter cells supported the formation of large syncytia after co-transfection with HSV-1 gB, gD, gH and gL. Within syncytia, the staining of nuclei with EYFPNuc could be detected by fluorescence microscopy. Fluorescence staining allows for the detection of small syncytia that are hardly visible microscopically, as, e.g., caused by the HSV-1 fusion machinery with wildtype gB-1.

### 3.3. HSV-1 Glycoprotein Mediated Cell–Cell Fusion Results in SEAP Reporter Gene Expression

**Figure 4:**
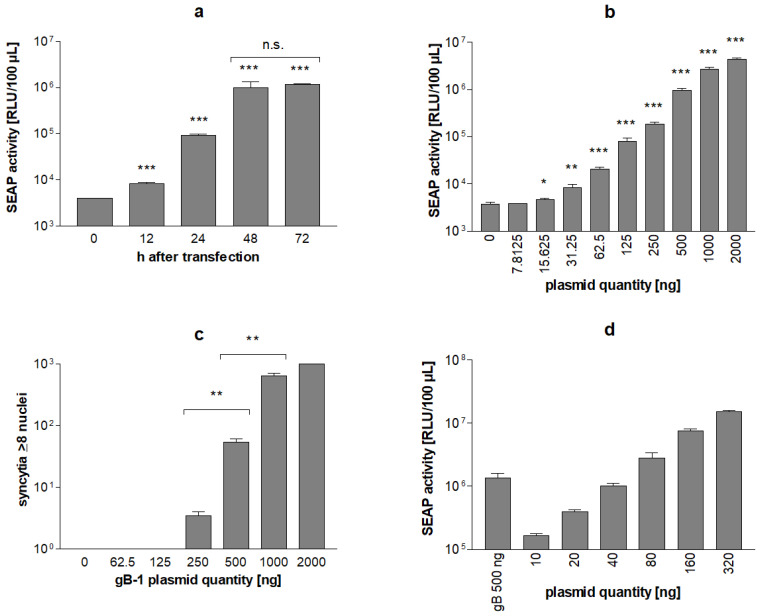
Syncytia formation by HSV-1 fusion machinery in Vero reporter cells induces SEAP expression. Time kinetics of SEAP expression are shown in panel (**a**). 2 × 10^5^ reporter cells cultivated in 24-well plates were transfected with gB-1 (500 ng), and gD-1, gH-1, and gL-1 plasmid (660 ng each), SEAP levels were quantified 12 to 72 h after transfection as indicated. The effect of gB-1 levels on SEAP expression is shown in panel (**b**). Cells were transfected with varying amounts of gB-1 plasmid as indicated and fixed amounts of gD-1, gH-1 and gL-1 (660 ng each), SEAP levels were quantified 48 h after transfection. Microscopical quantification of syncytia formation in reporter cells is shown in panel (**c**). Reporter cells cultivated in 24 well plates were transfected with fixed amounts of gD-1, gH-1, and gL-1 (660 ng each) and varying concentrations of gB-1 as indicated, fixed 48 h after transfection, stained with Giemsa and the number of syncytia with ≥8 nuclei was determined microscopically. Comparison of SEAP levels induced by syncytia formation and by direct transfection of indicator cells with the SEAP reporter plasmid is shown in panel (**d**). Reporter cells were either transfected with 500 ng gB-1 plasmid and 660 of gD-1, gH-1 and gL-1 plasmid each or with varying amounts of pTRE3G-SEAP-EYFPNuc as indicated. Data represent means ± SD from quadruplicates. Significance is indicated compared to 0 ng plasmid values, *p* < 0.05 (*), *p* < 0.01 (**) and *p* < 0.001 (***).

Quantification of SEAP levels in reporter cells transfected with the HSV fusion machinery at different time points showed that HSV-1 induced cell–cell fusion results in the secretion of SEAP into culture supernatants. SEAP secretion started around 12 h after transfection. Between 12 h and 48 h after transfection, SEAP activity strongly increased and reached peak levels around 48 h after transfection and remained stable on the following day (Figure 4a).

Transfection of Vero reporter cells with fixed amounts of HSV-1 gD, gH, gL and serial dilutions of gB-1 resulted in dose-dependent SEAP expression. The lowest amount of gB-1 plasmid DNA causing significant SEAP expression 48 h after transfection of 2 × 10^5^ reporter cells was 15.6 ng gB-1 plasmid. Transfection of 2000 ng gB-1 plasmid caused an approximately 1000-fold increase in the SEAP-specific chemiluminescence signal (Figure 4b). At gB-1 plasmid concentrations above 1000 ng, saturation of the SEAP response became evident. The dynamic range of the HSV-1 stable reporter fusion assay (SRFIA) was estimated to be 3 log_10_ orders.

As compared to the visual determination of syncytia formation in Giemsa-stained cultures (Figure 4c), the quantification of cell–cell fusion by HSV-1 SRFIA was found to be at least 5-fold more sensitive and to have an approximately 5-fold broader dynamic range.

As an internal positive control for HSV-1 SRFIA, the induction of SEAP expression by transfecting 500 ng gB-1 was compared to the effect of serial dilutions of pTRE3G-SEAP-EYFPNuc into the reporter cells. SEAP levels induced by 500 ng gB-1 corresponded to the direct transfection of approximately 40 ng pTRE3G-SEAP-EYFPNuc into reporter cells (Figure 4d).

### 3.4. Effect of Known Inhibitors of HSV-1 Cell–Cell Fusion on Reporter Gene Expression

The suitability of the HSV-1 SRFIA to detect the effects of known inhibitors of HSV-1-induced membrane fusion was evaluated by adding docosanol or the gB-specific neutralizing monoclonal antibody 2c, respectively, to cultures immediately after transfection.

**Figure 5:**
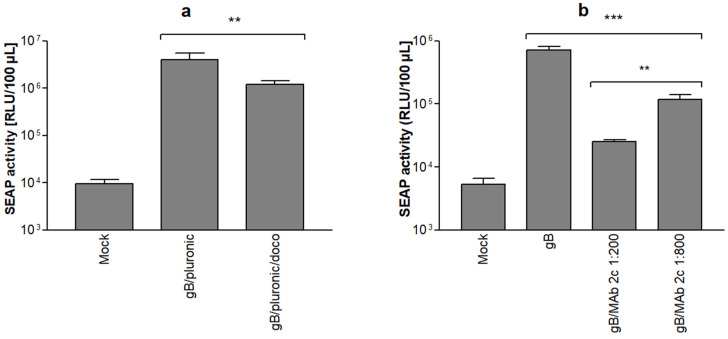
Effect of known inhibitors of HSV-1 glycoprotein-induced membrane fusion on SEAP levels. The inhibitory effect of docosanol is shown in panel (**a**). Reporter cells were treated with either 5 mg/mL docosanol/pluronic or 5 mg/mL pluronic only after transfection as indicated, SEAP levels were determined 48 h post transfection, Mock: empty gB-1 vector backbone. The inhibitory effect of a gB-specific, HSV-neutralizing monoclonal antibody (MAb 2c) is shown in panel (**b**). Reporter cells were treated with different concentrations of monoclonal antibody 2c after transfection as indicated, SEAP levels were determined 48 h post transfection, Mock: empty gB-1 vector backbone, no addition of monoclonal antibody. Data represent means ± SD from quadruplicate, *p* < 0.01 (**) and *p* < 0.001 (***).

As shown in Figure 5a, the treatment of cells with 5 mg/mL docosanol solubilized using the nonionic surfactant pluronic resulted in a significant, approximately four-fold reduction in the SEAP signal 48 h after transfection as compared to controls incubated with the solubilized pluronic only, which in itself enhances fusion. This is consistent with former investigations on the antiviral properties of docosanol, indicating an inhibitory effect in the fusion of the virus envelope and cell membrane by its metabolites [35,38]. Reduction in cell–cell fusion by docosanol was also evident in Giemsa-stained cultures. When evaluated microscopically, the cytotoxic effects of docosanol were not visible in Giemsa-stained cultures (Figure S1).

In addition, we analyzed the effect of the gB-specific, highly neutralizing monoclonal antibody 2c (MAb 2c) on cell–cell fusion. MAb 2c reduced SEAP levels in culture supernatants in a dose-dependent manner (Figure 5b). Notably, the inhibition of HSV-1 induced cell–cell fusion by MAb 2c was found to be highly effective. Even at the highest dilution tested (1:800), SEAP levels decreased by approximately 85%.

### 3.5. Establishment of Dual secNL/SEAP SRFIA

**Figure 6:**
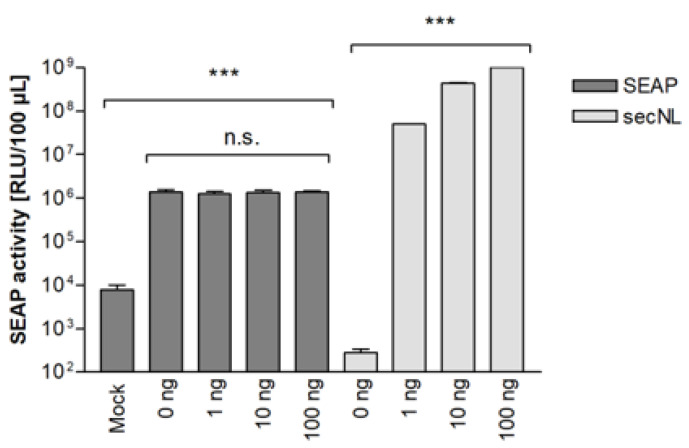
Co-expression of secNLuc as dual reporter system in SRFIA has no effect on SEAP levels. The effect of co-transfection of reporter cells with secNLuc on SEAP levels is shown. Reporter cells cultivated in 24-well plates were co-transfected with HSV-1 glycoproteins (500 ng gB-1, 660 ng gD-1, gH-1, and gL-1 each) and varying amounts of plasmid pEN-secNL as indicated, mock: empty gB-1 vector backbone. 48 h after transfection, secNLuc and SEAP levels were quantified in culture supernatants. Mock: empty gB-1 vector backbone. Data represent means ± SD from quadruplicate, n.s. not significant, *p* < 0.001 (***).

The cytotoxic effects of test compounds and variations in transfection efficiency, respectively, may lead to the false assessment of the inhibitory activity of compounds in SRFIA. As an additional reporter system sensitive to these systematic errors, we evaluated the co-transfection of the HSV-1 fusion machinery with a constitutively expressed form of the nanoluciferase (NLuc) secreted into culture supernatants (secNLuc). Due to its high enzymatic activity, the transfection of low amounts of the secNLuc expression plasmid resulted in high secNLuc activity in culture supernatants. Co-transfection of indicator cells with 1 to 100 ng secNLuc plasmid DNA had no effect on SEAP expression induced by the HSV-1 fusion machinery (Figure 6).

### 3.6. Performance of HSV-1-Specific SRFIA as Screening Assay for Fusion Inhibitors on 96-Well Plates

To facilitate the high-throughput screening (HTS) of potential fusion inhibitors, we adapted the HSV-1 SRFIA to a 96-well plate format. Of the several experimental approaches tested, the best results regarding reproducibility were obtained by co-transfecting the reporter cell mix grown in 6-well plates with the HSV-1 fusion machinery and secNLuc, resuspending and seeding transfected cells into 96-well plates, and incubating cultures for an additional 48 h prior to the quantification of the respective SEAP levels (for details see Materials and Methods). To determine signal separation between the no fusion protein plasmid control and positive fusion control, three SD values of the obtained values were calculated. The difference between data bands was at least 2 log_10_ orders (Figure S4a), indicating a sufficient signal-to noise ratio in a 96-well format.

**Figure 7:**
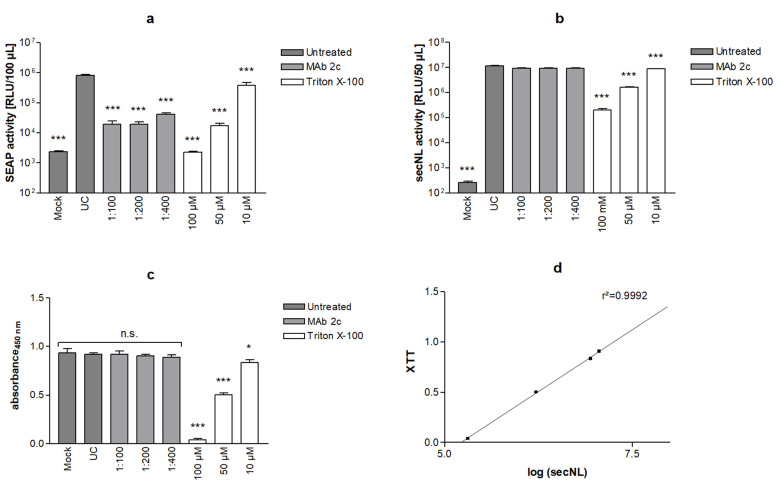
HSV-1 SRFIA on 96-well plates. Reporter cells cultivated on 6-well plates were co-transfected with vectors for gB-1 (500 ng), gD-1, gH-1, gL-1 (each 660 ng) and secNLuc (1 ng), transferred to 96-well plates and treated with the gB-1 specific monoclonal antibody MAb 2c or Triton X-100 as indicated. Untreated cells (UC, pos. fusion control) and cells transfected with empty gB-1 vector backbone (Mock, neg. fusion control) served as controls. 48 h after transfection, levels of SEAP (**a**) and secNLuc (**b**) were quantified in culture supernatants, and cell viability was determined by XTT assay (**c**). Significance is indicated compared to UC. Data represent means ± SD from quadruplicate. (**d**) correlation of secNLuc activity with XTT values, *p* < 0.05 (*), *p* < 0.001 (***).

The ability of the dual SEAP/secNLuc reporter HSV-1 SRFIA in 96-well plates to detect specific fusion inhibitors was evaluated by comparing the inhibitory effects of HSV-1 gB-specific monoclonal antibody 2c as the positive control with Triton X-100 as the cytotoxic test compound. SEAP and secNLuc levels in culture supernatants were determined as described above and the metabolic activity of cultures was assessed using the XTT assay. As expected, SEAP expression was strongly reduced by the inhibition of cell–cell fusion by monoclonal antibody 2c (Figure 7a). In addition, SEAP activity in culture supernatants was found to be highly sensitive to the Triton X-100 treatment of cultures. The treatment of cells with 10 µM Triton X-100 only moderately reduced SEAP levels, whereas the addition of 50 µM and 100 µM Triton X-100 led to a strong decrease in SEAP levels. At 100 µM Triton X-100, SEAP levels did not differ from background levels in untreated controls transfected with empty gB-1 vector backbone pBacMam-2. The Triton X-100 effect on SEAP levels corresponded well with cell morphology. Treatment with 100 µM Triton X-100 led to the complete disruption of cultures, whereas 10 µM Triton X-100 did not impact cell morphology (Figure S2).

In contrast to SEAP activity, secNLuc activity was not affected by the inhibition of cell–cell fusion with MAb 2c, however, it was strongly reduced by the treatment of cells with 100 and 50 µM Triton X-100 (Figure 7b).

The XTT assay was used to assess cell metabolism based on formazan salt formation by the reduction of a tetrazolium ring, providing the advantage of a water-soluble reaction product over the traditional MTT cell viability assay. The determination of cytotoxic effects by the XTT assay showed that the treatment of cells with MAb 2c had no effect on XTT staining, whereas 50 µM and 100 µM Triton X-100 caused a strong decrease in cell viability (Figure 7c). A direct comparison of XTT staining and the secNLuc signal demonstrated an excellent correlation of both assays (Figure 7d); however, due to the rapid expression of secNLuc after transfection, the signal was not reduced to zero.

To assess the ability of SRFIA to detect specific hits when used for the screening of compound libraries, Zhang indices (ZI) [37] of SEAP values as shown in Figure 7 were calculated (Table 1). ZI indices of negative fusion controls vs. positive fusion controls, and MAb 2c treated cultures vs. positive fusion controls were above 0.5, confirming that the separation band of the HSV-1 specific SRFIA is suitable for HTS.

### 3.7. SARS-CoV-2 S Protein Induces Formation of Syncytia and SEAP Expression in Reporter Cells

**Figure 8:**
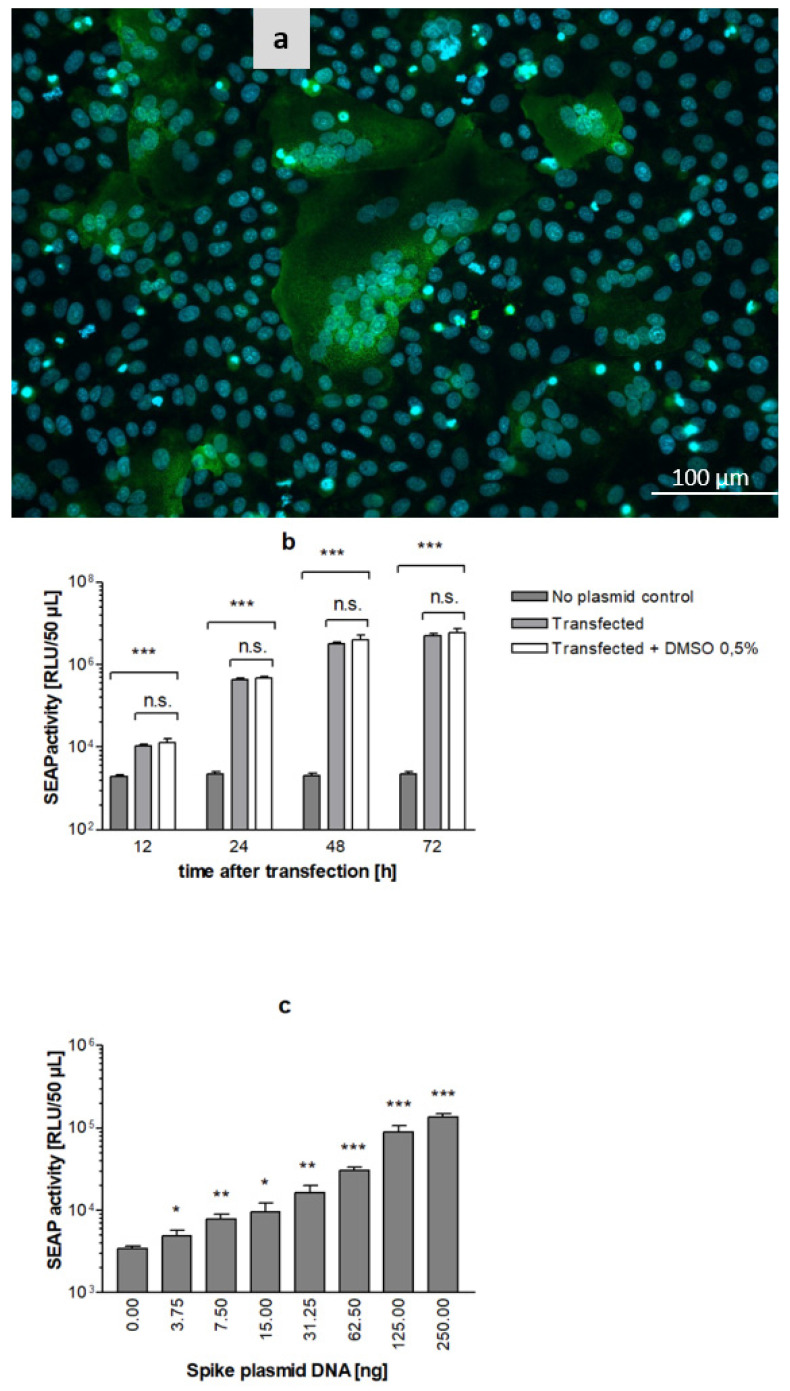
SARS-CoV-2 S protein induces syncytia formation and reporter gene expression in stable Vero reporter cells. (**a**) Reporter cells were transfected with 250 ng vector pCG1-SARS-2-S, fixed 24 h after transfection with formaldehyde 3.7%, and stained with SARS-CoV-2 IgG positive human serum/goat anti human IgG-FITC (green) and DAPI (blue); (**b**) time kinetics of SEAP-expression in SARS-CoV-2 transfected reporter cells cultivated in 24-well plates. Addition of DMSO 0.5% after transfection of cells (white bars) had no effect on SEAP levels; (**c**) Effect of plasmid quantity on SEAP activity measured 24 h post transfection. Significance is indicated compared to negative control. Data represent means ± SD from quadruplicate, *p* < 0.05 (*), *p* < 0.01 (**) and *p* < 0.001 (***).

Transfection of the SARS-CoV-2 S protein in the absence of the virus has been reported to induce cell–cell fusion in Vero E6 cells [24]. We thus tested the ability of SRFIA to detect and quantify cell–cell fusion induced by the S protein. The transient transfection of stable Vero reporter cells with S protein induced the formation of large syncytia (Figure 8a), and secretion of SEAP into culture supernatants. SEAP levels peaked between 48 h and 72 h after the transient transfection of reporter cells. DMSO was added and values were compared to untreated samples to determine if DMSO was suitable as a vehicle for test substances. The addition of DMSO 0.5% did not influence SEAP expression (Figure 8b). Comparable to HSV-1 SRFIA, the dynamic range of SARS-CoV-2 SRFIA was estimated to be approximately 3 log_10_ orders. SEAP levels corresponded in a dose-dependent manner to serial dilutions of the SARS-CoV-2 S expression vector (Figure 8c). The lowest amount of S protein plasmid causing significant fusion activity above the background level was 3.75 ng.

A total of 250 ng plasmid DNA was shown to be suitable for the high and robust expression of SEAP, whereas higher amounts of plasmid led to decreased reporter expression due to reduced cell viability.

### 3.8. Effect of Umifenovir on Syncytia Formation by the S Protein

Umifenovir (Arbidol) is a drug authorized in Russia for influenza treatment and has been described as an inhibitor of SARS-CoV-2 S protein-mediated cell–cell fusion in vitro, probably interacting with viral entry and intracellular vesicle trafficking [39]. In the reporter assay, the addition of 10 and 20 µM umifenovir after transfection resulted in SEAP signals being reduced to approximately 10 and 2% of the untreated control, respectively (Figure S3), without eliciting visible cytopathic effects at 10 and 20 µM. The addition of 50 µM umifenovir reduced SEAP levels to background activity. Treatment with 50 µM umifenovir, however, resulted in visible cytotoxicity, which is in accordance with a published CC_50_ of 31,79 µM [39].

To determine the assay specificity, camostat mesylate, which has been shown to inhibit TMPRSS2 protease and thus SARS-CoV-2 entry in lung epithelial cells [7], was tested. Since TMPRSS2-mediated membrane fusion does not play a role in Vero cell entry, no effect on cell–cell fusion and reporter enzyme expression was expected. This was confirmed by the obtained results (Figure S5).

### 3.9. Performance of S Protein-Specific SRFIA as Screening Assay on 96-Well Plates

**Figure 9:**
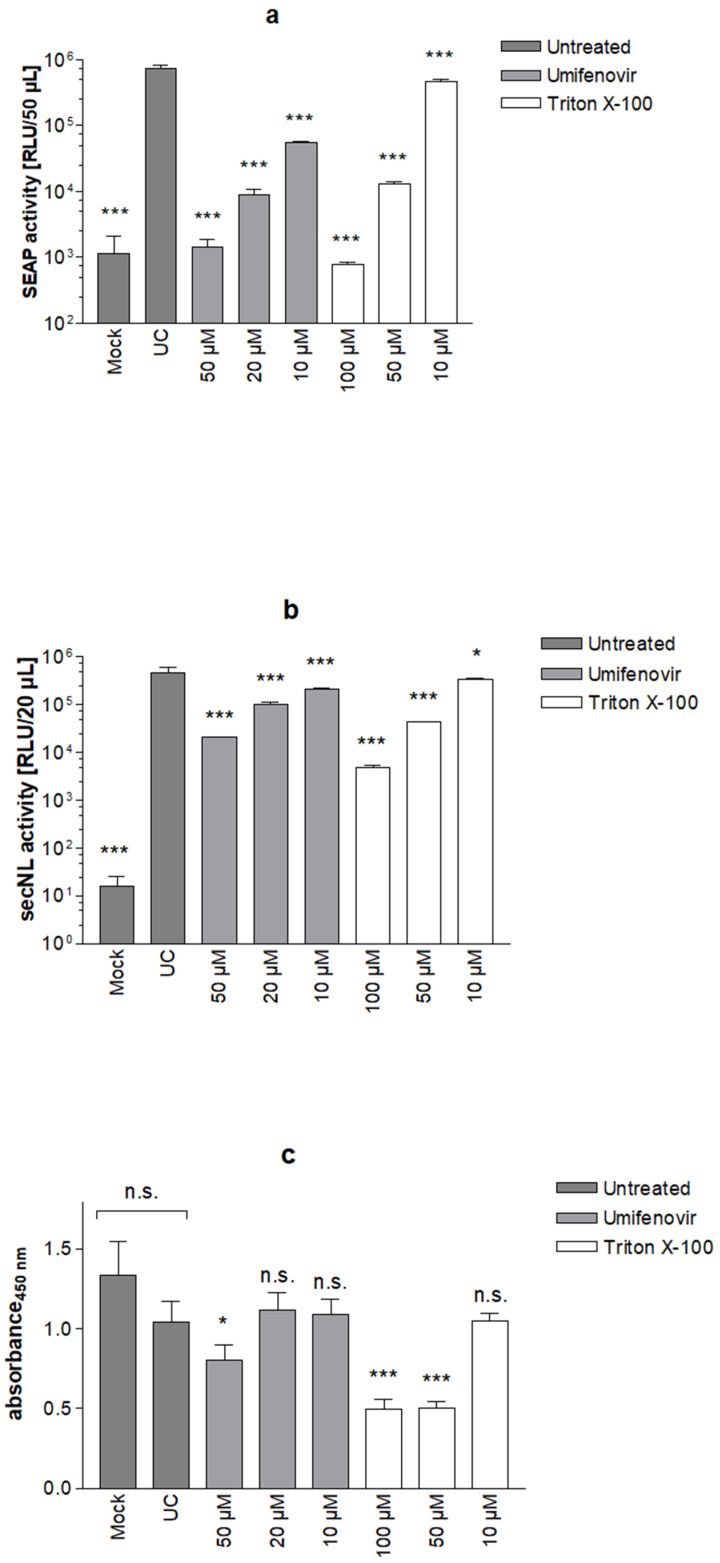
SARS-CoV-2 SRFIA on 96-well plates. Fusion assay on 96-well plates with umifenovir (10, 20, and 50 µM) or Triton X-100 detergent (10, 50, 100 µM) treatment, measurement was performed after 48 h of incubation; (**a**) SEAP levels, (**b**) secNLuc levels, (**c**) XTT values. Mock: no S protein/no secNLuc plasmid, no treatment. Significance is indicated compared to untreated control (UC). Data represent means ± SD from triplicate, n.s. not significant, *p* < 0.05 (*) and *p* < 0.001 (***).

Finally, we evaluated the performance of the S protein-specific SRFIA on 96-well plates using the dual reporter approach as described for the HSV-1 specific SRFIA (Figure 9a–c). 3 SD data variability bands of positive fusion control and negative control were separated by at least 2 log_10_ orders, comparable to the results for HSV-1 (Figure S4b). Treatment with 10, 20 and 50 µM umifenovir and 50 and 100 µM of the cytotoxic control Triton X-100, respectively, significantly reduced SEAP levels in a dose-dependent manner (Figure 9a). Levels of secNLuc in culture supernatants were almost unchanged in cells treated with 10 µM umifenovir, indicating a specific, fusion-reducing effect of umifenovir on S-induced cell–cell fusion in SRFIA. Higher concentrations of umifenovir resulted in a stepwise decrease in secNLuc levels, most likely due to the initiation of cytotoxic effects. A significant reduction in secNLuc levels were also observed with the cytotoxic control Triton X-100 added at a concentration of 50 µM and 100 µM, whereas the addition of 10 µM Triton X-100 had no significant effect on secNLuc levels (Figure 9b). In Triton X-100 treated cells, the results of secNLuc correlated well with the outcome of the XTT assay (Figure 9c). Triton X-100 (50 and 100 µM) strongly reduced cellular catabolic activity, while 10 µM led to slight effects on secNL levels and no significant effect on XTT values. As compared to secNLuc levels, umifenovir appeared to have less of an effect on the respective XTT values, e.g., the addition of 10 and 20 µM umifenovir did not reduce XTT values significantly, whereas 50 µM of this compound resulted in a moderate reduction in XTT values.

Analogous to HSV-1, the correlation between secNLuc and Triton X-100 concentration was excellent (R^2^ = 0.99, data not shown), whereas the correlation between XTT and secNLuc was not as precise as shown for the HSV-1 assay, due to a lack of sensitivity of the XTT assay.

Zhang indices for the negative control and umifenovir-treated samples compared to the untreated control were calculated (Table 2). All values were above 0.5, thus, the assay was rated as suitable for screening on 96-well plates.

## 4. Discussion

Reporter fusion assays are useful tools for the analysis and quantification of virus-mediated membrane fusion. Built up as a virus-free approach, they greatly facilitate laboratory workflow by avoiding handling infectious viruses. The aim of our study was to establish a robust, sensitive, and universally applicable stable reporter fusion inhibition assay (SRFIA) with the focus on the identification of antifusogenic compounds. The following, important basic test attributes were taken into account when establishing the SRFIA: (i) strictly fusion-specific induction of reporter gene expression, (ii) simple and highly sensitive quantitation of reporter gene expression, (iii) ability to support multiple measurements without the need to lyse indicator cells, (iv) minimal number of experimental steps, and (v) avoidance of expensive laboratory equipment.

Based on these criteria, we decided to use doxycycline-dependent reporter gene expression. Originally described by Gossen et al. (1992) [40], the third-generation doxycycline-inducible Tet-On 3G system provides highly specific gene expression in cell culture and laboratory animals with reduced background expression and increased sensitivity to doxycycline [41]. To achieve fusion-specific activation of the reporter gene, the basic components of the Tet-On 3G system, i.e., the Tet3G activator and the TRE3G responsive element, can be placed separately into effector and target cells. Since in vivo experiments revealed no immune responses after retinal application [42], adverse effects on target cells such as initiating an innate immune response using the Tet-On 3G system were expected to be negligible in the cell culture. Promising experiments using Tet-On 3G for in vivo immunotherapy have been conducted [43], providing an increase in the safety and specificity of therapies. This contrasts with other approaches used in reporter fusion assays such as T7-polymerase/T7-promoter-based reporter gene expression, which has been described to trigger unwanted innate immune responses [44].

Reporter gene SEAP was chosen, which can be detected by chemiluminescence with a sensitivity of up to 10-fold higher compared to firefly luciferase (FLuc) [45]. Secretion into culture supernatants provides multiple measurements of SEAP activity without the need to lyse indicator cells [46]. The nuclear localization of EYFPNuc was found to be helpful in determining the size of syncytia and for cell sorting when selecting stable indicator cells.

As indicator cells, a pair of stable effector and reporter cell lines containing the individual components of the fusion-dependent Tet-On 3G reporter gene expression system was generated. The use of stable indicator cell lines circumvents the problem of a varying transfection efficiency of reporter genes, which can only partly be standardized.

Vero E6 cells were chosen as stable effector and reporter cells. Due to the deletion of alpha and beta-1 interferon genes, Vero cells support the replication of many viral pathogens including SARS-CoV-2 [7,47] and are of importance for vaccine production [48]. The formation of syncytia using the selective expression of viral fusogens was observed with members of various families of enveloped viruses such as filoviruses, alphaviruses, paramyxoviruses, coronaviruses and alphaherpesviruses (reviewed by [10,49,50]). The use of stable effector and target cells differing solely in the components of the reporter system facilitates the direct transfection of the pre-seeded indicator cell mix with viral fusogens, thereby reducing the number of experimental steps by avoiding different pretreatments of target and effector cells. Following pre-screening in stable Vero E6 indicator cells, the transient transfection of the reporter system into target cells might allow for the study of antifusogenic compounds in cells more closely resembling natural host cells.

As a defined chemical compound inhibiting HSV-1-mediated membrane fusion, docosanol was applied as the positive control. Docosanol cream has been approved for the topical treatment of reactivated herpes simplex [51]. Poor hydrophilicity requires the addition of docosanol to cultures such as opaque 10% suspension in 10% pluronic as the solubilizer [35]. To obtain pronounced antiviral effects in tissue culture, cells must typically be pretreated with docosanol for 24 h or longer [35]. In our system, the pretreatment of indicator cells with docosanol suspension was omitted to avoid unwanted effects on transfection. Notably, docosanol was correctly identified as a fusion inhibitor by SRFIA despite the relatively short duration of treatment and the presence of residual docosanol suspension during the quantitation of SEAP activity.

Umifenovir was applied as the test compound in SARS-CoV-2-specific SRFIA. Although clearly cytotoxic at higher concentrations, a reduction in SARS-CoV-2-S-mediated cell–cell fusion by umifenovir was observed at concentrations within the therapeutic range in patients. It was thus concluded that the SRFIA was principally able to detect chemical compounds known to interfere with virus-induced membrane fusion [35,36,39,52].

To tailor SRFIA specifically to the needs of compound screening, several additional controls were implemented. The expression of viral fusogens in indicator cells by transient transfection is a critical step, which cannot be fully standardized. In the case of highly sensitive reporter assays, a dual reporter approach, i.e., transfection of a second, constitutively expressed chemiluminescent reporter, proved successful [53]. Regarding our approach, the co-transfection of the constitutively expressed secreted reporter secNLuc was found to effectively facilitate the detection of both reporters in culture supernatants. Our data show that secNLuc and SEAP do not cross-react in culture supernatants and that the co-expression of secNLuc with the HSV-1 fusion machinery does not impede syncytia formation. The very high specific activity of NLuc [54] holds true also for secNLuc and allows for the co-transfection of low concentrations of the secNLuc reporter plasmid, i.e., 1 ng per 2 × 10^5^ cells, thereby further reducing unwanted effects on fusogen expression and syncytia formation. A similar approach using two secreted reporters, e.g., *Gaussia* luciferase (GLuc) and *Cypridina* luciferase (CLuc), was described by Wu et al. 2007 [55,56]. In the case of SEAP and NLuc, both reporters demonstrated glow chemiluminescence and were highly stable during incubation and in culture supernatants [45,53], providing long incubation periods with the storage of culture fluids at −20 °C before quantification.

The reliable detection of cytotoxic effects of test compounds and/or solubilizing agents is essential to identify and eliminate false positive hits in SRFIA. Thus, the inclusion of cytotoxicity controls is indispensable. The defined induction of cytotoxic effects in cell culture can be achieved with various approaches and compounds differing in their mode of action, time kinetics, extent of cell damage and cell specificity [57,58]. Since cell–cell fusion in SRFIA starts within hours following transfection, the treatment of cells with Triton X-100 resulting in rapid cell lysis was found to be a suitable cytotoxicity control.

Frequently, cell viability is quantified by measuring the metabolic activity of cells, e.g., by MTT or XTT assays [58]. Our data show that the dual SEAP/secNLuc reporter system provides the sensitive detection of Triton X-100-mediated cytotoxic effects without the need for cell lysis, facilitating repeated measurements from the same sample, and correlates well with the outcome of XTT assay performed in parallel. To detect relatively weak or delayed cytotoxic effects, the expression of secNLuc from a different, less active promoter, e.g., by replacing the CMV IE promoter by a tk promoter, should be considered.

As a positive control for HSV-1 SRFIA, an HSV-1 gB-specific monoclonal antibody with potent neutralizing activity and strong inhibition of cell–cell fusion even at high dilutions was used. The absence of cytotoxic effects of monoclonal antibodies renders them well suited for use as positive inhibition controls. This also indicates the suitability of the assay to determine the effectivity of neutralizing antibodies, which can be of special interest in serological screenings and clinical studies as well as in drug discovery, especially in cases of the fast development of the resistance of viral strains against authorized antibody therapies, e.g., the SARS-CoV-2 omicron variant [59].

Downsizing SRFIA to a 96-well or 384-well plate format, respectively, is essential for the screening of compound libraries or fractionated natural substances. Our results demonstrate that SRFIA performs well on 96-well plates and provides the reliable identification of specific hits, i.e., of compounds inhibiting HSV-1 and SARS-CoV-2-mediated fusion. In both cases, a ZI of >0.5 indicated excellent suitability for compound screening [37]. To rate sensitivity in a 384-well format, further research is needed.

Using SRFIA to quantify the inhibition of membrane fusion by chemical compounds, several methodical characteristics and limitations must be considered. Of note, SRFIA does not allow for the real-time detection of the membrane fusion process itself, which requires different experimental approaches (e.g., [15,60,61]). In addition, apart from virus-induced membrane fusion itself, the formation of syncytia involves and depends on a multitude of steps such as maturation and the intracellular transport of viral fusogens, virus receptors, function and structure of cell contacts, cytoskeleton and lipid membranes [62]. Compounds primarily affecting these host cell factors may also be detected as specific hits by SRFIA. As long as the inhibition of these steps is not associated with relevant cytotoxicity, these ‘off-target’ effects may represent valuable hits [63]. Finally, there is still room for further methodical improvements of SRFIA, as described here. The establishment of stable effector cell lines with a regulated expression of viral fusogens may simplify the workflow, improve reproducibility and allow further downsizing to a 384-well plate format. In addition, the pretreatment of cultures with compounds prior to the induction of cell–cell fusion may be facilitated. While the establishment of cell lines expressing the complex, quadripartite fusion machinery of HSV-1 requires complex strategies, the use of stable cell lines constitutively expressing SARS-CoV-2-S protein in SRFIA has already been described [17]. On the other hand, transiently transfecting viral fusogens in SRFIA can assist in the rapid adaptation of the assay to epidemiologically relevant events, e.g., novel variants of concern of SARS-CoV-2 or other emerging pathogens, thus providing additional advantages.

In conclusion, the SRFIA described here represents a robust, highly sensitive and reproducible approach for the identification of compounds specifically interfering with HSV-1- and SARS-CoV-2-induced membrane fusion. The assay can be performed with routine laboratory equipment, is suitable for HTS and should be expandable to other viruses due to its modular design. In particular, the secreted dual reporter system enables the simultaneous detection of fusion inhibition and cytotoxic side effects at multiple time points without the prior lysis of cells.

## Figures and Tables

**Table 1:** Zhang indices of HSV-1-specific SRFIA on 96 well plate calculated based on at least *n* = 8 values each.

Sample	Zhang Index
untreated vs. negative control	0.89
untreated vs. anti-gB 1:100	0.65
untreated vs. anti-gB 1:200	0.72
untreated vs. anti-gB 1:400	0.74

**Table 2:** Zhang indices for SARS-CoV-2 SRFIA on 96-well plate. Values are calculated from at least *n* = 6 values.

Sample	Zhang Index
untreated vs. mock control	0.94
untreated vs. umifenovir 50 µM	0.87
untreated vs. umifenovir 20 µM	0.78
untreated vs. umifenovir 10 µM	0.66

## Data Availability

Not applicable.

## Supplementary Materials

The following supporting information can be downloaded at: https://www.mdpi.com/article/10.3390/v14071354/s1, Figure S1: Treatment of reporter cells with pluronic or pluronic/docosanol, Figure S2: Effect on cell morphology by Triton X-100 on Vero cells, Figure S3: Inhibition of SARS-CoV-2 S mediated cell–cell fusion by the known inhibitor umifenovir on 24-well plates, Figure S4: Data variability bands of the SRFIA for HSV-1 and SARS-CoV-2, Figure S5: Effect of camostat mesylate on SARS-CoV-2 SRFIA as non-fusion inhibiting control.
