# Supplementary material for: Broadly Applicable, Virus-Free Dual Reporter Assay to Identify Compounds Interfering with Membrane Fusion: Performance for HSV-1 and SARS-CoV-2
Source: Viruses. 2022 Jun 21;14(7):1354. doi: 10.3390/v14071354 (PMC9322530; doi:10.3390/v14071354)
Supplement: Supplementary file 1 [file viruses-14-01354-s001.zip › viruses-1737090-supplementary.pdf]

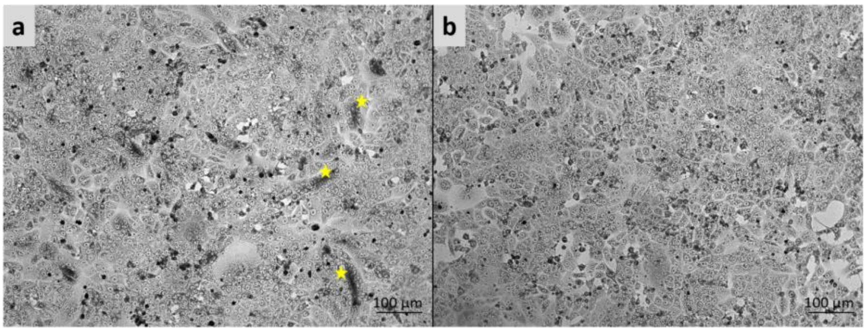

Figure S1. Treatment with pluronic or pluronic/docosanol. Vero E6 cells were transfected with HSV-1 fusion machinery and treated with (**a**) pluronic 5 mg/mL or (**b**) pluronic/docosanol 5 mg/mL each. After 24 h of incubation, cells were stained with Giemsa staining solution. Syncytia are exemplarily marked with yellow asterisks.

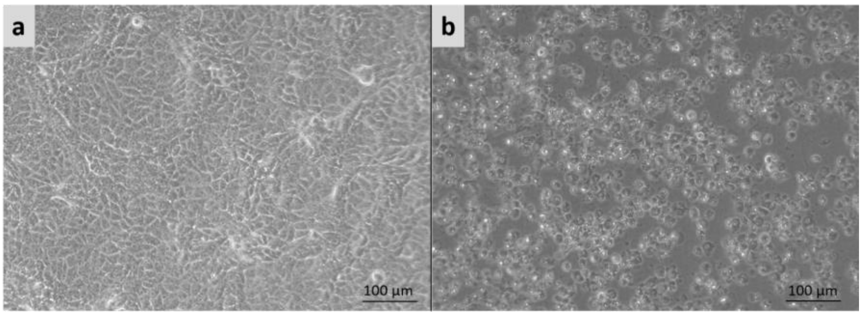

Figure S2. Effect of Triton X-100 on Vero cells. 2 × 10^5 Vero cells cultivated on a 24-well plate were incubated with (**a**) 10 µM or (**b**) 100 µM Triton X-100 for 24 h to verify cytotoxic effects.

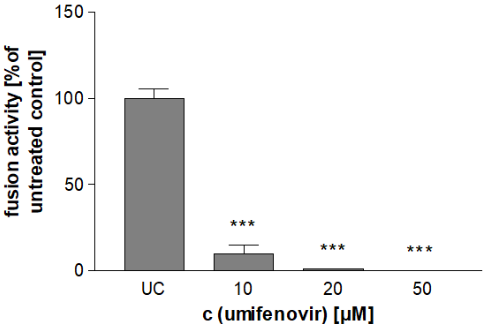

Figure S3. Inhibition of SARS-CoV-2 S mediated cell–cell fusion by the known inhibitor umifenovir on 24-well plates. Untreated control containing 0.5% DMSO serves as 100% fusion activity control. Measurement was performed 24 h post transfection. Significance is indicated compared to the untreated control (UC). Data represent means ± SD of two independent experiments, each performed in technical triplicate.

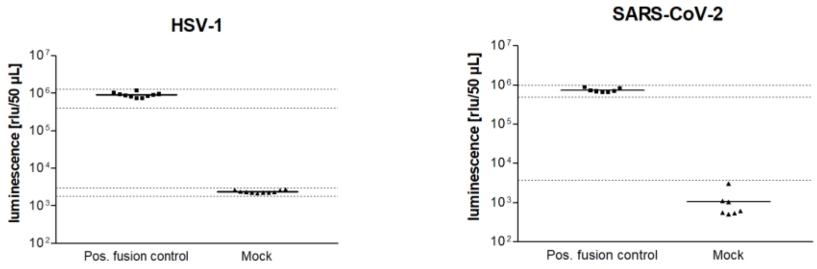

Figure S4. Data variability bands of the SRFIA for HSV-1 (**a**) and SARS-CoV-2 (**b**). Data variability is indicated for positive (Pos.) fusion control and negative control (Mock). Every dot represents one value obtained from measurement on 96-well plates after 48 h of incubation. Solid and dotted lines indicate mean ± 3 SD values, calculated from n=10/n=7 samples, respectively.

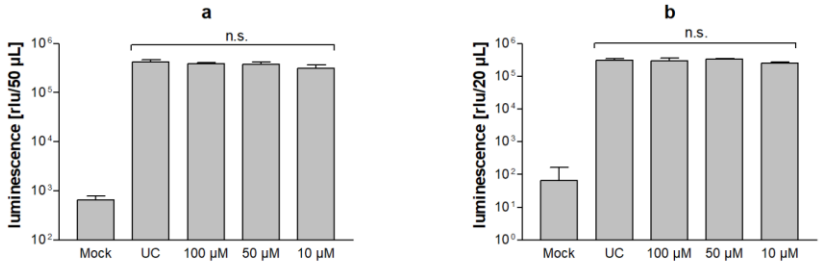

Figure S5. Effect of camostat mesylate on SARS-CoV-2 S SRFIA as fusion non-inhibiting control. Measurements of SEAP (**a**) and secNLuc (**b**) activity were performed after 48 h of incubation on 24-well plates. Data represents means ± SD from triplicates. Significance is indicated compared to untreated control (UC) containing DMSO as vehicle control.
